# Analysis of Corticosteroid-Induced Glaucoma Using the Japanese Adverse Drug Event Reporting Database

**DOI:** 10.3390/ph16070948

**Published:** 2023-06-30

**Authors:** Ayano Kawabe, Yoshihiro Uesawa

**Affiliations:** Department of Medical Molecular Informatics, Meiji Pharmaceutical University, Tokyo 204-8588, Japan

**Keywords:** glaucoma, corticosteroids, intraocular pressure, Japanese Adverse Drug Event Report (JADER) database, spontaneous reporting system, volcano plot, hierarchical clustering, principal component analysis

## Abstract

Glaucoma is the most common cause of blindness, which significantly reduces quality of life. Most glaucoma cases are primary glaucoma; nevertheless, many patients suffer from glaucoma caused by drugs, such as corticosteroids. A comprehensive review of the risks associated with corticosteroid-induced glaucoma is limited. Therefore, we used the Japanese Adverse Drug Event Reporting Database (JADER) published by the Pharmaceuticals and Medical Devices Agency (PMDA) to analyze the risk factors associated with glaucoma and the trends and characteristics of corticosteroid-induced glaucoma. We did not find sex or age differences associated with the onset of glaucoma. Hierarchical clustering and principal component analysis revealed that triamcinolone acetonide and betamethasone sodium phosphate, which are used around the eyes in Japan, are more likely to induce intraocular pressure (IOP) elevation compared with other corticosteroids. Increased IOP is a direct cause of glaucoma. Based on these findings, it may be necessary to limit or avoid the use of these corticosteroids.

## 1. Introduction

In Japan, the most common cause of visual impairment among people over 60 years old is glaucoma (28.6%) [1], which glaucoma has increased in recent years. Visual impairment significantly decreases patient quality of life. There are a wide variety of glaucoma types, but secondary glaucoma is caused by diseases (including systemic diseases) and drugs have been reported along with primary glaucoma. According to the Tajimi study, the prevalence of confirmed total glaucoma cases over the age of 40 is 5.0% (95% CI, 4.2–5.8), whereas secondary glaucoma, which is a rare type, accounts for 0.3% (95% CI, 0.13–0.51) of the confirmed cases [2,3].

Drug-induced glaucoma is primarily caused by corticosteroids. Adrenergic agonists, cholinergics, and anticholinergics can induce glaucoma; however, it is rare and the underlying mechanism is different from that of corticosteroids [4]. The use of corticosteroids induces IOP elevation, which increases the risk of developing glaucoma. People with elevated IOP caused by corticosteroid use are known as corticosteroid responders [5]. There are individual differences in IOP elevation [5]. In the normal population, approximately 61–63% are nonresponders to corticosteroids, exhibiting IOP elevations < 5 mmHg, 33% exhibit a moderate increase in IOP (6–15 mmHg), and 4–6% are high responders to corticosteroids (IOP elevation > 15 mmHg). Furthermore, children under the age of 10 show marked IOP elevation caused by corticosteroids [5].

Several clinical trials have demonstrated that corticosteroids induce glaucoma; however, a comprehensive analysis of glaucoma-inducing tendencies by corticosteroid type has not been conducted. This comprehensive analysis provides an effective treatment strategy that minimizes the risk of glaucoma during corticosteroid use. Additionally, it investigates unexplored aspects of corticosteroid types and routes of administration, thereby improving glaucoma-induced risk assessment. Therefore, using the Japanese Adverse Drug Event Report database (JADER), a database of spontaneous reports of suspected side effects published by the Pharmaceuticals and Medical Devices Agency (PMDA), we analyzed the risk of glaucoma caused by corticosteroids and their characteristics.

## 2. Results

### 2.1. Preparing Data Tables for Analysis

**Figure 1:**
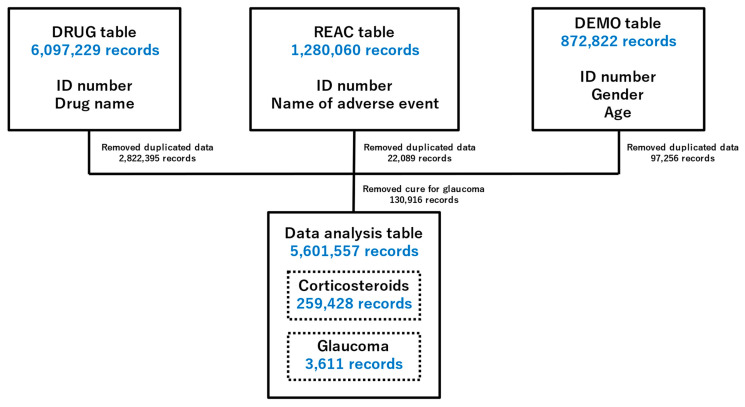
Flowchart for the construction of the data analysis tables. DRUG, drug information; REAC, adverse reaction information; DEMO, patient demographic information. Duplicate data in the DRUG, REAC, and DEMO tables were deleted. Three tables were combined using identification numbers. Patients taking glaucoma medications were removed from the analysis.

Figure 1 shows a flowchart for creating a data table for analysis. The data were extracted from the drug information (DRUG) table (6,097,229 records), adverse reaction information (REAC) table (1,280,060 records), and patient demographic information (DEMO) table (872,822 records) from JADER. We removed cured glaucoma patients, eliminated three duplicate tables, and combined them, resulting in 5,601,557 records in a final data table. The data table for analysis included 3611 and 259,428 records for glaucoma and corticosteroid-related cases, respectively.

### 2.2. Relevance of Glaucoma to Patient Characteristics

Table 1 shows the characteristics of patients with glaucoma. In the glaucoma patient group, we did not detect any association with sex or age.

### 2.3. Relationship between Glaucoma and Drugs

**Figure 2:**
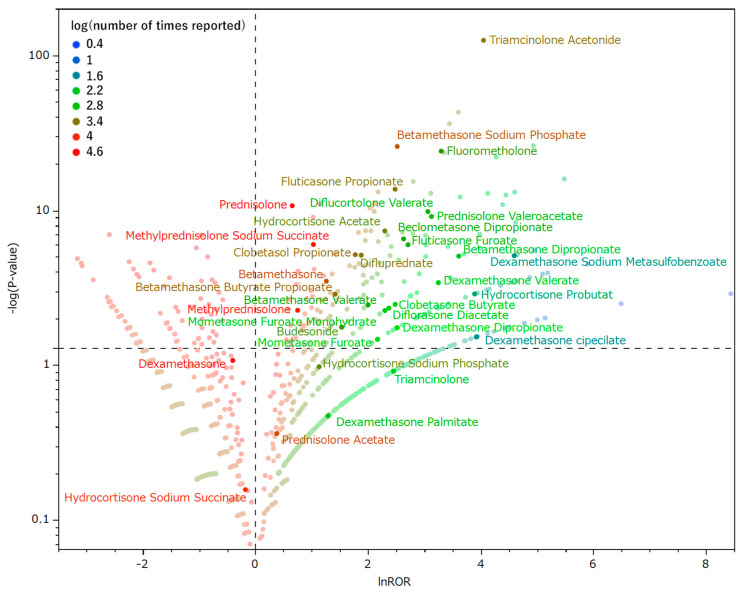
Drugs associated with glaucoma. The X-axis shows the natural logarithm of the reporting odds ratio (lnROR). The Y-axis shows the reciprocal of the common logarithm for the *p*-value of Fisher’s exact test [−log(*p*-value)]. RORs were calculated using a crosstabulation table. The dotted line on the Y-axis represents a *p*-value of 0.05. The color of the plot represents the number of reported adverse events. The common logarithm of the total number of reported times is shown in red to blue.

Figure 2 shows the association of glaucoma with drug use. Drugs plotted in the upper right tend to induce glaucoma. The labeled drugs are corticosteroids.

Table 2 shows the reporting odds ratios for the association between corticosteroids and glaucoma.

### 2.4. Hierarchical Clustering of Glaucoma-Inducing Corticosteroids

**Figure 3:**
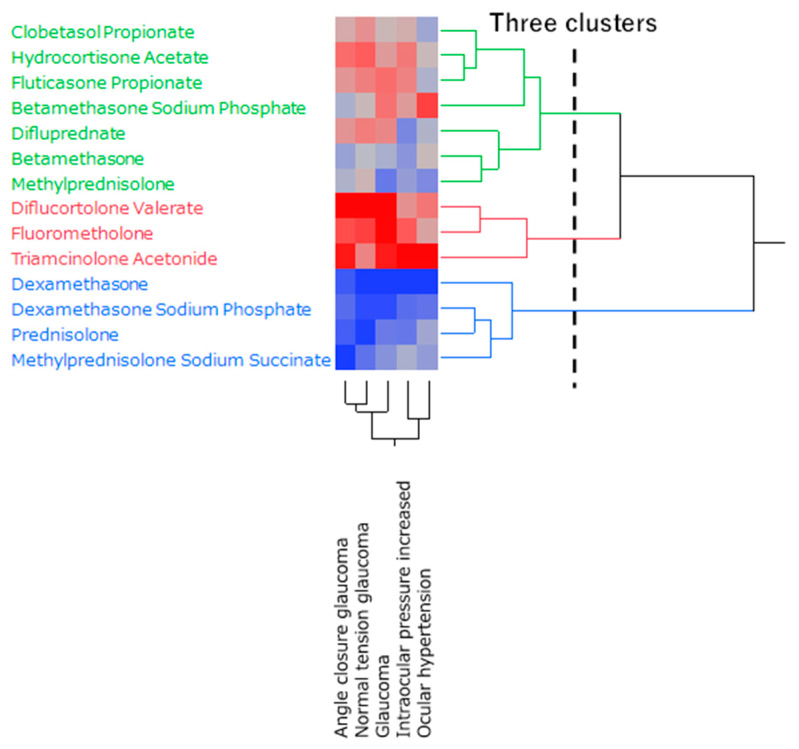
Classification of corticosteroids associated with glaucoma using hierarchical clustering. The dendrogram shows the relationship between 14 corticosteroids and five glaucoma-related side effects. The color map shows the correlation between the variables.

Figure 3 shows the dendrogram generated by hierarchical clustering resulting in two clusters.

Table 3 shows the number of corticosteroid reports used for hierarchical clustering.

### 2.5. Principal Component Analysis of Glaucoma-Induced Corticosteroids

**Figure 4:**
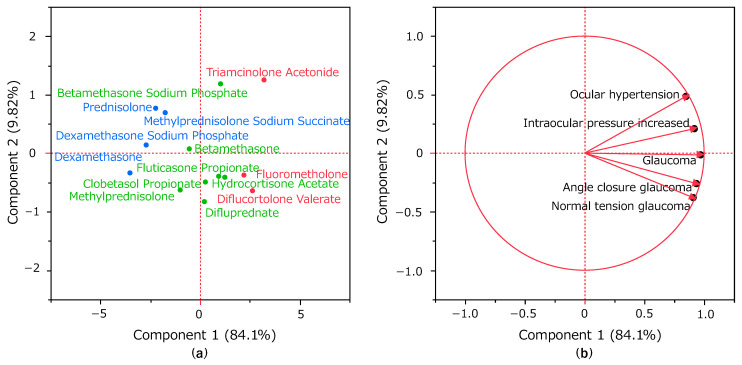
Association between glaucoma and corticosteroids using principal component analysis. Principal component score plot (**a**) shows the relationship between corticosteroids and each principal component. Each plot represents a corticosteroid. The color of each plot represents classification by hierarchical clustering. Principal component loading plot (**b**) shows the relationship between glaucoma-related adverse events and each principal component. Each loading vector represents an adverse event.

Figure 4 shows the results of the principal component analysis. The contribution of the principal components was 84.1% for principal component one and 9.82% for principal component two. We created a scattergram using principal components one and two. We then visualized the relationship between glaucoma-related adverse events and each principal component representing each adverse event as a loading vector. The X-axis represents principal component one, which was positively correlated with the ROR. The Y-axis represents principal component two, ocular hypertension (0.48487), and intraocular pressure increased (0.20892) exhibited a positive correlation. Normal tension glaucoma (−0.38005), angle-closure glaucoma (−0.25989), and glaucoma (−0.01468) showed a negative correlation.

## 3. Discussion

### 3.1. Glaucoma and Characteristics of the Patient

In this study, we observed no significant differences in incidence of glaucoma based on sex and age.

Malclès et al. found that men have a 2.24-fold increased risk of IOP elevation with corticosteroid administration compared with women (multivariate analysis, 95% CI: 1.38–3.63, *p*-value < 0.001) [6]. On the other hand, the Rotterdam study showed that the earlier the age of menopause (i.e., the shorter the duration of exposure to female hormones), the higher the risk of open-angle glaucoma [7]. The results of the two previous studies differ from those obtained in the present study. Both previous studies are epidemiological studies of primary glaucoma. Since JADER is a large database of adverse drug reaction reports, registered glaucoma is an adverse event resulting from drug treatment. In other words, unlike primary glaucoma, drug-induced glaucoma may not show significant sex differences.

The present study also revealed that there was no difference between the two groups at either age 70 and over or age 40 and over. Choi et al. reported that the rate of IOP elevation after intravitreal injection of dexamethasone, a type of corticosteroid, was 42.9% for those aged 30 years or younger, 35.3% for those aged 31–40 years, 28.3% for those aged 41–50 years, 14.9% for those aged 51–60 years, 12.2% for those aged 61–70 years, 8.4% for those aged 71–80 years, and 9.1% for those aged 81–90 years. The incidence of IOP elevation was reported to increase with younger age [8]. In addition, ages below 30 years are a risk factor for IOP elevation, according to Parekh et al. [9]. In contrast, Garbe et al. showed that the administration of oral corticosteroids to elderly patients was associated with a 1.41-fold (95% CI, 1.22–1.63) increased risk of IOP elevation or open-angle glaucoma compared with the untreated group [10]. Thus, different results have been reported in previous studies. We believe that the reason for this discrepancy is the insufficient number of subjects enrolled in these clinical studies; however, the large database analysis used in this study did not show statistically significant differences in age despite the extremely large number of subjects.

### 3.2. Glaucoma and Corticosteroids

In this study, we identified 28 corticosteroids that were statistically significantly associated with inducing glaucoma. Hierarchical clustering showed that corticosteroids were divided into three clusters. Thereafter, the principal component analysis revealed that the three clusters were correlated with principal component one, indicating lnROR, and principal component two, indicating easy induction of IOP elevation. Triamcinolone acetonide and betamethasone sodium phosphate were observed to induce IOP elevation.

Corticosteroids are well-known drugs that cause glaucoma [11]. Therefore, the analysis in the present study focused on corticosteroids. Of the 47 corticosteroids in clinical use as prescription drugs in Japan (excluding combination drugs and discontinued products, only those reported to JADER) [12], 28 were plotted on the upper right in the volcano plot [−log(*p*-value) > 1.3 and lnROR > 0]. Thus, it was estimated that these corticosteroids tended to induce glaucoma significantly (Figure 2). Corticosteroids, which are associated with a low number of glaucoma reports, are a confounding factor and do not provide the differences in characteristics between corticosteroids that we would expect to observe and that tend to induce glaucoma.

Corticosteroid-induced glaucoma has symptoms similar to primary open-angle glaucoma [13]. Although open-angle glaucoma is the most common type of glaucoma among the Japanese population, only 42 cases of open-angle glaucoma were reported in JADER. The lowest level term of the preferred term “Glaucoma” includes “corticosteroid glaucoma”, “steroid-induced glaucoma”, “secondary glaucoma”, etc. In other words, corticosteroid-induced glaucoma is not reported by type, but is registered collectively as the preferred term “glaucoma”. Therefore, contrary to the expectation that open-angle glaucoma would be the most frequently reported type of corticosteroid-induced glaucoma, we presumed that there were fewer reports of open-angle glaucoma.

Hierarchical clustering, which groups similar data, divided corticosteroids into three clusters based on preferred terms related to glaucoma (Figure 3). The cluster (red) that exhibited a strong correlation with all five glaucoma-related preferred terms included three corticosteroids (diflucortolone valerate, fluorometholone, and triamcinolone acetonide). The cluster showing a relatively weak correlation (green) included seven corticosteroids (clobetasol propionate, hydrocortisone acetate, fluticasone propionate, betamethasone sodium phosphate, difluprednate, betamethasone, methylprednisolone). Four corticosteroids (dexamethasone, dexamethasone sodium phosphate, prednisolone, and methylprednisolone sodium succinate) that showed a weak correlation (blue) all had more than 10,000 records (Table 3). Based on these results, a principal component analysis was performed to further clarify the characteristics of each corticosteroid.

Principal component analysis may be used to summarize and visualize the bias of explanatory variables [14]. Color coding based on the three clusters obtained in the hierarchical cluster analysis indicated a correlation with principal component one. Furthermore, principal component one was strongly correlated with lnROR, indicating that it is readily induced for glaucoma (Figure 4). In contrast, principal component two in Figure 4b, which shows the principal component loadings, was plotted in the positive direction for ocular hypertension and intraocular pressure increased, and in the negative direction for glaucoma, angle-closure glaucoma, and normal tension glaucoma. Both ocular hypertension and intraocular pressure increase are preferred terms, indicating a condition of IOP elevation. Glaucoma may be caused by IOP elevation [15], but not all glaucoma cases are necessarily associated with IOP elevation [16]. Therefore, principal component two may indicate a simple induction of IOP elevation. Although a negative correlation was observed between the use of adrenal corticosteroids and certain types of glaucoma in our study, it does not mean that there is little or no relationship between steroid use and angle-closure glaucoma. In particular, the mechanism and development of angle-closure glaucoma are strongly associated with aging, and considering the inclusion of a recognizable elderly population in JADER [17], further clinical trials are necessary to draw definitive conclusions. Figure 4a shows the score plot. This indicates corticosteroids with high values for principal component one, which indicates easy induction, and positive values for principal component two, which indicates IOP elevation associated with triamcinolone acetonide and betamethasone sodium phosphate. These two corticosteroids were presumed to readily induce IOP elevation. Triamcinolone acetonide is the only corticosteroid administered intravitreously in Japan for vitreous visualization during vitrectomy and for the reduction of macular edema, such as diabetic macular edema. The intravitreal injection of triamcinolone acetonide readily induces IOP elevation. Roth et al. found that in eyes injected with intravitreous triamcinolone acetonide (929 eyes in 841 patients), the incidence of IOP elevation above 21 mmHg two years after injection was 44.6% (95% CI, 41–50%) [18]. Betamethasone sodium phosphate is used in Japan as an eye, ear, and nose drop. Topical administration of corticosteroids, such as eye drops, is associated with a higher risk of glaucoma compared with systemic administration [19]. The results of the present study support this clinical finding. Corticosteroids with high values for principal component one and negative values for principal component two included clobetasol propionate, diflucortolone valerate, difluprednate, hydrocortisone acetate, fluorometholone, and fluticasone propionate. These corticosteroids are associated with a smaller tendency to induce IOP elevation compared with other corticosteroids. Fluorometholone is an eye drop, fluticasone propionate is a nasal drop, and the others are transdermal drugs, such as ointments and creams. Transdermal drugs, even in the periocular area, have a low risk of causing glaucoma when administered over short periods [20], which is consistent with the present findings. We found that corticosteroids may have different rates of side effects depending on the method of administration. This may lead to the development of new methods, such as changing the route of administration to avoid glaucoma and IOP elevation in patients requiring treatment with corticosteroids.

### 3.3. Limitations

There are several limitations to this study. First, there are limitations with respect to the database used. Since this database contains information on adverse reactions based on spontaneous reports, cases are limited to those that are recognized as adverse reactions. Specifically, mild side effects may occur occasionally, whereas severe side effects may be frequent. This is known as reporting bias, a characteristic of self-reporting databases [21]. In this study, the total number of patients who used corticosteroids could not be ascertained, so an accurate assessment of the adverse events was not possible. Therefore, we tried to increase the usefulness of the analysis by setting a filter for the number of reports and avoiding easy *p*-value and ROR comparisons. Second, some JADER data were incomplete and may contain blank cells, such as missing values or incorrectly entered letters or numbers. When missing values for sex and age were found, they were removed and addressed. Third, when multiple drugs are administered, it is difficult to identify the specific cause of adverse events. Although fatal adverse events registered in JADER are confirmed by PMDA, other adverse events are based on the reporter’s judgment and may include not only actual adverse events but also questionable adverse events. Nonetheless, JADER is the largest database of voluntary reports for adverse drug reactions in Japan. Adverse drug reaction information obtained from JADER is expected to reflect pharmacological and pharmacokinetic properties and prescribing and usage conditions. Therefore, JADER is an excellent tool for understanding adverse drug reactions and may be used across several research areas. Fourth, this analysis is a risk assessment of patients who reported adverse reactions in Japan. Therefore, the results cannot be directly applied to the general population. Hence, more detailed additional studies that consider differences in patient backgrounds, such as race and history of the disease, are necessary.

## 4. Materials and Methods

### 4.1. Database (JADER)

JADER is an adverse drug reaction database published by PMDA that summarizes suspected adverse drug reactions reported by manufacturers, distributors, or medical institutions. The data used in the present study were downloaded from the PMDA website [22], ranging from 1 April 2004 to 30 June 2022. Since this study used anonymized data from an open-access database, the requirements for ethical approval and informed consent were waived by the Ethics Committee of Meiji Pharmaceutical University.

### 4.2. Drugs to Be Analyzed and Adverse Event Terms

The drugs analyzed were corticosteroids, which are currently used clinically in Japan. Combination drugs were excluded from the analysis [12]. Glaucoma was defined by MedDRA/J version 25.0 [23]. Of the 47 narrow-scope terms of standardized MedDRA queries (Table 4), the preferred terms (32) excluding congenital, traumatic, postoperative, and surgical terms were analyzed (Table 5).

### 4.3. Creation of a Data Table for Analysis

The analysis was performed using the DRUG, REAC, and DEMO tables of JADER. Three operations were performed using these tables. First, when “;” was included in the route, date, dose, dosage unit, and several divided doses in DRUG, the lines were stacked for each “;”. Second, when “;” was included in the reporter information in the DEMO, the lines were stacked for each “;.” Third, if the drug name was a combination drug, the rows were stacked by the “·” in the name. The completed tables were used for analysis. Duplicate data were deleted in each of the three tables and joined by identification number. Patients taking glaucoma medications were removed from the data tables.

### 4.4. Glaucoma and Characteristics of the Patients

The Tajimi study represents an epidemiological study of glaucoma conducted among people aged 40 years or older living in Tajimi City, Gifu Prefecture, Japan. The results indicated that 5.0% (95% CI, 4.2–5.8) of those aged 40 years or older had glaucoma [2,3]. Therefore, an analysis was conducted in two separate segments: age 40, which is generally considered to have a sharply increased risk of glaucoma, and age 65 and older, defined as elderly by the World Health Organization (in the 70s for age-specific reporting in JADER). Age was grouped by age group. Elderly people in their 70s, 80s, and 90s and elderly people were defined as “age 70 and older.” In addition, individuals in their 40s, 50s, 60s, 70s, 80s, 90s, and the elderly were defined as “age 40 and older”. Adults and unknown subjects were not included in the analysis. They were further divided into two groups according to the presence or absence of glaucoma, and a *p*-value was calculated using a bivariate relationship to examine the presence of a significant difference. The *p*-value was calculated using Fisher’s exact test. The analysis was performed only on data that did not contain missing values.

### 4.5. Glaucoma and Corticosteroids

The association between 47 corticosteroids and glaucoma was analyzed. The risk of glaucoma on corticosteroids was assessed using the ROR and Fisher’s exact test. First, a crosstabulation table (Table 6) was created, in which cells with zero cannot be calculated. If the cell value is small, the estimation becomes unstable. Therefore, 0.5 was added to all cells as a correction (the Haldane correction) [24]. Drugs with a ROR of one or higher and a *p*-value of less than 0.05 in Fisher’s exact test were considered glaucoma-inducing corticosteroids [25]. Next, to visualize adverse events, a scatterplot containing ROR and *p*-values was created. This scatterplot used ROR as the natural logarithm (lnROR) and the *p*-value obtained from Fisher’s exact test as the common logarithm of the reciprocal. Scatterplots correspond to volcano plots and are often used to visualize gene expression trends [26,27].

### 4.6. Hierarchical Clustering

Of the 47 corticosteroids (Table 4), only 14 were reported in 100 or more cases, and 10 or more reported glaucoma cases were included. In addition, only five preferred terms for glaucoma were included in 50 or more reported preferred terms. We calculated the ROR from the crosstabulation table, converted the resulting ROR to the natural logarithm, and performed hierarchical clustering to objectively classify them. The Ward method based on Euclidean distance with loads from five preferred terms was used [28].

### 4.7. Principal Component Analysis

Similar to hierarchical clustering, only 14 corticosteroids were reported in 100 or more cases and 10 or more reported glaucoma cases were included and only five preferred terms with 50 or more reports were included. The ROR was calculated from the crosstabulation table (Figure 3), and it was converted to a natural logarithm. Principal component analysis was performed using the covariance matrix and focused on principal components one and two.

### 4.8. Statistical Analysis

Statistical analysis was performed using JMP Pro16 software (SAS Institute Inc., Cary, NC, USA). The level of statistical significance was set at 0.05.

## 5. Conclusions

Analysis of JADER data revealed a trend in corticosteroids that cause glaucoma, a rare side effect. All corticosteroids for which effective values were obtained in JADER were shown to induce glaucoma. Furthermore, different types of corticosteroids exhibited different inducing IOP elevations, which can cause glaucoma. Triamcinolone acetonide and betamethasone phosphate sodium had a high tendency to cause IOP elevation. We anticipate that the results of this study will contribute to improved risk management for drug-induced glaucoma patients.

## Figures and Tables

**Table 1:** Characteristics of glaucoma and nonglaucoma patients.

	Patient Background	Glaucoma (1405)	Nonglaucoma (1,288,906)	*p*-Value (Fisher’s Exact Test)
Sex	Male	653/1347	599,030/1,209,010	0.445
	Female	694/1347	609,980/1,209,010	
Age	≥70 years old	436/1145	468,717/1,170,976	0.184
	<70 years old	709/1145	702,259/1,170,976	
	≥40 years old	949/1145	960,658/1,170,976	0.488
	<40 years old	196/1145	210,318/1,170,976	

**Table 2:** Reporting odds ratio of corticosteroids related to glaucoma.

Corticosteroids	Reporting Odds Ratio	95% Confidence Interval	*p*-Value
Alclometasone dipropionate	2.10	0.13–33.65	1.000
Amcinonide	43.99	2.65–731.68	1.000
Beclometasone dipropionate	13.90	7.07–27.33	<0.001
Betamethasone	3.53	2.02–6.15	<0.001
Betamethasone butyrate propionate	4.12	2.10–8.08	0.001
Betamethasone dipropionate	36.99	14.52–94.28	<0.001
Betamethasone sodium phosphate	12.41	8.95–17.22	<0.001
Betamethasone valerate	7.41	2.93–18.73	0.004
Budesonide	4.63	1.84–11.70	0.017
Ciclesonide	1.40	0.09–22.39	1.000
Clobetasol propionate	5.89	3.30–10.52	<0.001
Clobetasone butyrate	11.97	4.18–34.29	0.003
Cortisone acetate	12.32	0.76–199.16	1.000
Deprodone propionate	9.56	0.59–154.25	1.000
Dexamethasone	0.67	0.41–1.09	0.085
Dexamethasone cipecilate	50.77	9.98–258.32	0.030
Dexamethasone dipropionate	12.40	3.57–43.08	0.018
Dexamethasone palmitate	3.64	0.73–18.08	0.338
Dexamethasone sodium metasulfobenzoate	98.96	33.57–291.78	<0.001
Dexamethasone sodium phosphate	1.03	0.67–1.60	0.910
Dexamethasone valerate	25.74	8.95–74.07	<0.001
Diflorasone diacetate	10.71	3.74–30.66	0.005
Diflucortolone valerate	21.37	11.61–39.33	<0.001
Difluprednate	6.55	3.57–12.01	<0.001
Fludrocortisone acetate	2.04	0.13–32.67	1.000
Fludroxycortide	4.49	0.28–72.07	1.000
Fluocinolone acetonide	9.93	0.62–160.27	1.000
Fluocinonide	2.71	0.17–43.38	1.000
Fluorometholone	27.09	18.00–40.79	<0.001
Fluticasone furoate	15.03	7.32–30.88	<0.001
Fluticasone propionate	11.95	7.64–18.68	<0.001
Hydrocortisone acetate	9.95	5.57–17.79	<0.001
Hydrocortisone probutate	49.06	13.92–172.9	0.001
Hydrocortisone sodium phosphate	3.10	1.09–8.86	0.106
Hydrocortisone sodium succinate	0.84	0.36–1.93	0.696
Methylprednisolone	2.12	1.34–3.35	0.006
Methylprednisolone acetate	1.72	0.11–27.61	1.000
Methylprednisolone sodium succinate	2.80	1.97–3.98	<0.001
Mometasone furoate	8.74	2.52–30.32	0.034
Mometasone furoate monohydrate	9.98	3.49–28.56	0.006
Prednisolone	1.93	1.62–2.29	<0.001
Prednisolone acetate	1.46	0.63–3.38	0.435
Prednisolone sodium phosphate	4.17	0.26–66.97	1.000
Prednisolone sodium succinate	1.15	0.40–3.29	1.000
Prednisolone valeroacetate	22.78	12.00–43.27	<0.001
Triamcinolone	11.64	2.33–58.03	0.122
Triamcinolone acetonide	57.41	46.63–70.67	<0.001

**Table 3:** The number of times reported corticosteroids were used for hierarchical clustering.

Corticosteroids	Number of Times Reported
Clobetasol propionate	3023
Diflucortolone Valerate	768
Difluprednate	2485
Dexamethasone	37,816
Dexamethasone Sodium Phosphate	30,523
Triamcinolone Acetonide	2693
Hydrocortisone Butyrate	1794
Fluorometholone	1366
Fluticasone Propionate	2543
prednisolone	107,943
betamethasone	5483
Betamethasone Sodium Phosphate	4604
Methylprednisolone Sodium Succinate	17,429

**Table 4:** Analysis of 47 corticosteroids.

Corticosteroids	
Alclometasone Dipropionate	Amcinonide
Beclometasone Dipropionate	Betamethasone
Betamethasone Butyrate Propionate	Betamethasone Dipropionate
Betamethasone Sodium Phosphate	Betamethasone Valerate
Budesonide	Ciclesonide
Clobetasol Propionate	Clobetasone Butyrate
Cortisone Acetate	Deprodone Propionate
Dexamethasone	Dexamethasone cipecilate
Dexamethasone Dipropionate	Dexamethasone Palmitate
Dexamethasone Sodium Metasulfobenzoate	Dexamethasone Sodium Phosphate
Dexamethasone Valerate	Diflorasone Diacetate
Diflucortolone Valerate	Difluprednate
Fludrocortisone Acetate	Fludroxycortide
Fluocinolone Acetonide	Fluocinonide
Fluorometholone	Fluticasone Furoate
Fluticasone Propionate	Hydrocortisone Acetate
Hydrocortisone Probutat	Hydrocortisone Sodium Phosphate
Hydrocortisone Sodium Succinate	Methylprednisolone
Methylprednisolone Acetate	Methylprednisolone Sodium Succinate
Mometasone Furoate	Mometasone Furoate Monohydrate
Prednisolone	Prednisolone Acetate
Prednisolone Sodium Phosphate	Prednisolone Sodium Succinate
Prednisolone Valeroacetate	Triamcinolone
Triamcinolone Acetonide	

**Table 5:** Analysis of 32 preferred terms (MedDRA/J version 25.0).

Preferred Terms	Number of Reports
Acute myopia	0
Angle-closure glaucoma	507
Aphakic glaucoma	0
Borderline glaucoma	0
Diabetic glaucoma	0
Exfoliation glaucoma	1
Fundoscopy abnormal	21
Glaucoma	1790
Glaucoma drug therapy	0
Glaucomatocyclitic crises	16
Glaucomatous optic disc atrophy	1
Gonioscopy abnormal	0
Halo vision	0
Intraocular pressure fluctuation	2
Intraocular pressure increased	838
Intraocular pressure test abnormal	1
Loss of visual contrast sensitivity	1
Malignant glaucoma	4
Normal tension glaucoma	52
Ocular hypertension	272
Open-angle glaucoma	41
Optic discs blurred	1
The optical nerve cup/disc ratio increased	0
Optic nerve cupping	40
Phacolytic glaucoma	0
Pigmentary glaucoma	0
Pseudophakic glaucoma	0
Pupillary light reflex tests abnormal	7
Slit-lamp tests abnormal	0
Uveitic glaucoma	1
Uveitis-glaucoma-hyphaema syndrome	0
The visualVisual field tests abnormal	15

**Table 6:** A crosstabulation table and ROR formulas. A crosstabulation table consisting of reports with the suspected medicine, all other reports, reports of glaucoma, and reports of nonglaucoma.

	Glaucoma	Nonglaucoma
Reports with the suspected medicine	a	b
All other reports	c	d

ROR (Reporting Odds Ratio) = (a/b)/(c/d) = ad/bc.

## Data Availability

Data is contained within the article.
